# IMPDH2's Central Role in Cellular Growth and Diseases: A Potential Therapeutic Target

**DOI:** 10.1111/cpr.70031

**Published:** 2025-04-19

**Authors:** Zheng Li, Yunpeng Zou, Jiayao Niu, Ying Zhang, Aohua Yang, Wenyu Lin, Jie Guo, Shuya Wang, Ronghan Liu

**Affiliations:** ^1^ Central Hospital Affiliated to Shandong First Medical University, Shandong First Medical University and Shandong Academy of Medical Sciences Jinan China; ^2^ School of Clinical Medicine, Shandong Second Medical University Weifang China; ^3^ Department of Orthodontics School and Hospital of Stomatology, Cheeloo College of Medicine, Shandong University Jinan China

## Abstract

IMPDH2 is a rate‐limiting enzyme in guanine nucleotide biosynthesis. It plays diverse roles in various physiological and pathological processes: nucleotide metabolism, inflammation, immune function, ribosomal stress. Structural or regulatory alterations in IMPDH2 are linked to significant health issues, and critical relevance in disease progression. We aim to underscore the potential of IMPDH2 as a promising therapeutic target for clinical applications.

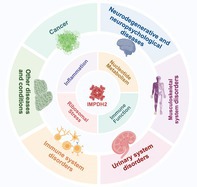

To the Editor,

Inosine 5′‐monophosphate dehydrogenase (IMPDH) is a rate‐limiting enzyme in guanine nucleotide biosynthesis, catalysing the NAD+‐dependent oxidation of inosine monophosphate (IMP) to xanthine monophosphate (XMP). IMPDH inhibition depletes the guanine nucleotide pool, reducing GTP levels and impairing cell proliferation, underscoring its critical role in DNA and RNA synthesis [1] (Figure 1). Two isoenzymes are present in human IMPDH, IMPDH1 and IMPDH2, a total of 514 amino acids, with 84% amino acid sequence similarity in amino acid sequence. The regulation of IMPDH activity is primarily mediated by IMPDH2, rather than IMPDH1 [3, 4]. IMPDH2, a prominent member of the IMPDH family, has gained the notable attention of researchers due to its diverse roles in cell proliferation, differentiation and chemoresistance [3]. IMPDH2 is universally expressed at the organ system level but has variable physiological expression levels within different tissues. Emerging research suggests that IMPDH2 has distinct functional roles in both physiological and pathologic states. Dysregulation of IMPDH2 has been confirmed by researches linked to a wide range of diseases. This letter will systematically expatiate the structure of IMPDH2 and the multiple intracellular functions of IMPDH2 in multiple conditions, in order to highlight the critical role of IMPDH2 in disease and its potential therapeutic role in the clinical practice.

**FIGURE 1:**
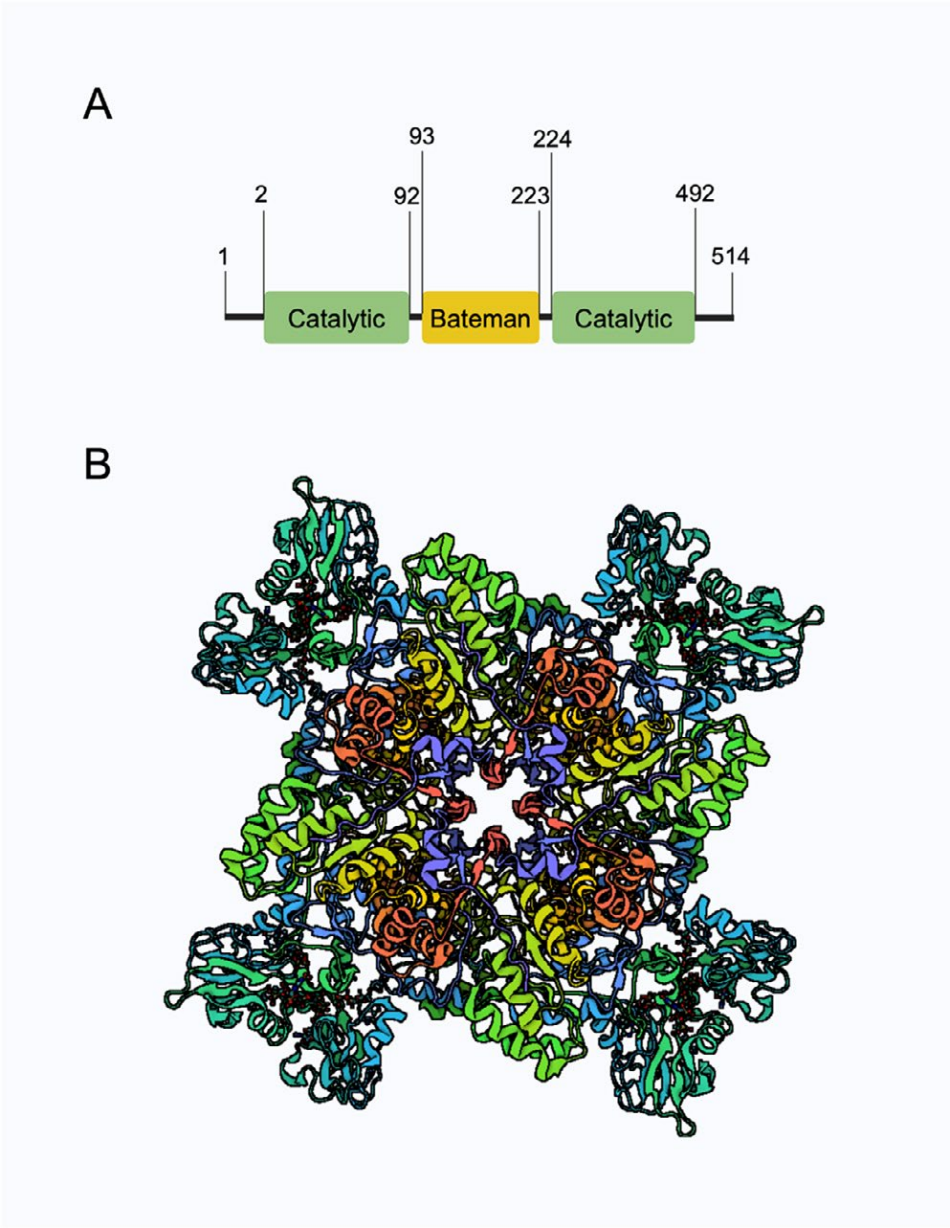
The molecular structure of IMPDH2. (A) IMPDH2 has a total 514 aminoacids, and consists of two domains: A catalytic domain (amino acid residues 2–92 and 224–492) forming the core of the active enzyme; and a regulatory Bateman domain (amino acid residues 93–223). Green represents the catalytic domain and yellow represents the bateman domain. (B) 3D rendering of a free standard octamer. *Source:* Data from https://www.rcsb.org/3d‐view/6UC2,viewed using pymol [2].

## The Structure of IMPDH2

MPDH2 is a free canonical octamer, with each monomer containing two structural domains: the catalytic structural domain **(amino acid residues 2–92 and 224–492)** for the conversion of IMP and NAD+ to XMP and NADH, and the Bateman regulatory structural domain [5] (Figure 1). The catalytic domain adopts a canonical (β/α)8 barrel fold. Similar to other proteins with this fold, the active site resides on a ring formed by the C‐terminus of the beta sheet. Containing the ring of catalytic Cys319, both the C‐terminal segment and a flexible loop (skin flap) exhibit varying degrees of conformational contingency upon the bound ligand. The C‐terminal fragment is coupled to the Cys319 ring via a monovalent cation. Crystallographic data suggest that IMPDH2 likely adopts distinct conformational states throughout the catalytic cycle [6]. The large segment between β8 and α8 forms a flap which occludes the active site. Like the Cys319 loop, this flap is disorganised to varying degrees depending on the ligand. Most dramatically, the distal portion of the flap moves in and out of the active site in the catalytic cycle; the dehydrogenase reaction requires an open conformation, whereas the hydrolysis step uses a closed conformation [7].

The substructural domain contains two CBS structural domains (named after homologous structural domains of cysteothionein synthase); also known as Bateman regulatory domains **(amino acid residues 93–223)**. The Bateman regulatory domain has three distinct binding sites for binding adenine nucleotides (A) and guanine nucleotides (G) [8, 9, 10], whereas site 3 exclusively binds GDP/GTP. CBS domains are not universally required for enzymatic activity, and certain IMPDH enzymes, including those from *B. burgdorferi* and *C. parvum* , lack CBS subdomains. Additionally, IMPDH2 interacts with nucleic acids [11], and this function is interfered with by deletions and mutations within the CBS domains [12]. In cultured cells, subdomains mediate IMPDH2 binding to polyribosomes, suggesting a role in translational regulation [13] which may also underlie the regulation of bacterial purine nucleotide pools.

The tetramer adopts a square planar geometry, with the barrel sides positioned at the subunit interface. The CBS subdomains protrude from the quadrants of the tetramer. The connection between the Bateman regulatory domains and catalytic domains is random, enabling relative orientations to vary by up to 120° in different crystal structures [14].

## The Biological Functions of IMPDH2

**FIGURE 2:**
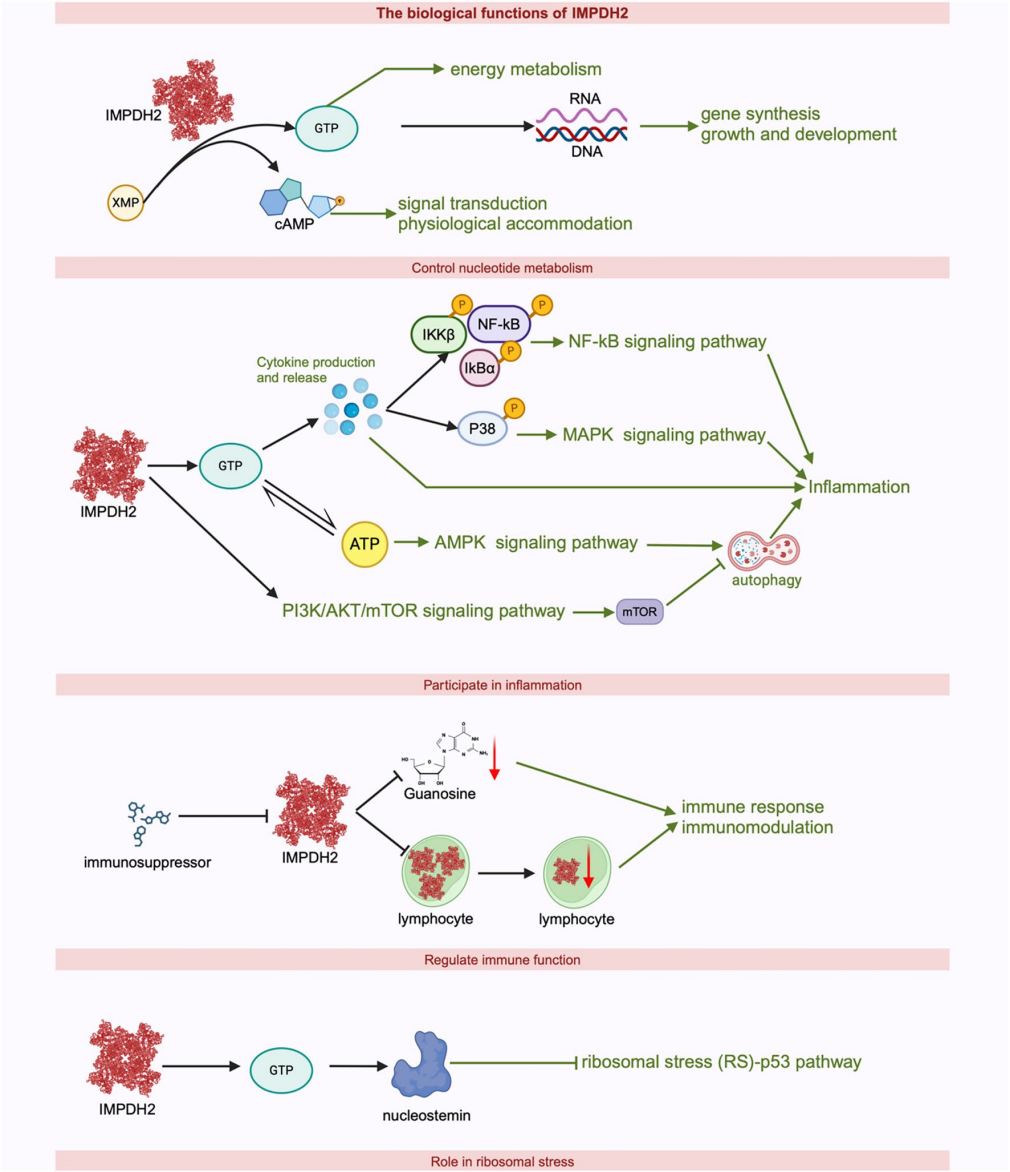
The biological functions of IMPDH2. (A) Participates in the synthesis of XMP to GTP. (B) Affect the energy supply and thus participate in inflammation signal transduction pathways. (C) Similar immunosuppressive agents that act specifically on IMPDH2, thus participating in the immune response and immunomodulation. (D) By inhibiting cellular IMPDH2, it reduces GTP levels, which in turn reduces nucleostemin (NS) levels, which in turn activates RS and p53 pathway.

IMPDH2 plays diverse roles in a variety of physiological and pathological processes, as illustrated in Figure 2. These functions are detailed below.

### Control Nucleotide Metabolism

IMPDH2 is a key enzyme at the branch point of adenine and guanine nucleotide synthesis metabolism and is essential for the de novo synthesis of guanine nucleotides (GTP). NAD+ is involved in the IMPDH‐catalysed dehydrogenation of inosine monophosphate (IMP) to xanthosine monophosphate (XMP), a precursor in GTP synthesis. ATP and GTP, crucial metabolites of IMPDH2 [15] fuel diverse cellular physiological processes, including material transport, muscle contraction and nerve transmission. These high‐energy phosphate compounds also play a pivotal role in anabolism and intracellular energy transfer.

Nucleotides are the basic units for the synthesis of ribonucleic acid (RNA) and deoxyribonucleic acid (DNA). Inhibition of intracellular IMPDH2 activity reduces GTP levels, consequently impairing DNA and RNA synthesis [1]. Nucleotides, as the building blocks of DNA, are directly involved in the DNA repair process. When DNA is damaged, the relevant enzymes in the nucleotide metabolic pathway are able to recognise and repair these damages, preserving the stability and integrity of the genome. Therefore, normal IMPDH2 activity is the basis for the transmission and expression of genetic information in organisms, as well as the maintenance of energy metabolism, growth and development.

In this process, cyclized nucleotides such as cAMP and cGMP are also produced as second messengers, which are involved in intracellular signalling and physiological regulation [16]. In summary, IMPDH2 plays a critical role in maintaining energy metabolism, growth and development, cellular signalling, biomolecule synthesis and organismal stability. More is still worth exploring and discovering.

### Participate in Inflammation

IMPDH2 participates in the inflammatory response by influencing the production and release of cytokines. Cytokines such as: NO, IL‐6 and TNF‐a are important signalling molecules in the inflammatory response. IMPDH2, as a key enzyme in purine nucleotide synthesis, not only produces a large amount of energy but also is accompanied by the formation of purine nucleotides. Their synthesis and release require a lot of energy, which is also inseparable from IMPDH2. In addition, purine nucleotides, as components of DNA or RNA, are involved in gene transcription and translation of cytokines. By screening small molecule compounds targeting IMPDH2, we found that inhibiting IMPDH2 markedly reduced GTP level and significantly reduced the release of inflammatory mediators such as IL‐6, NO and TNF‐α [17]. Alterations in IMPDH2 activity may directly or indirectly influence the expression of inflammation‐related genes and the production of inflammatory mediators.

By adjusting the function of IMPDH2, subsequently inhibiting inflammatory downstream signalling pathways [18, 19]. When IMPDH2 is inhibited, the GTP level also be inhibited. Activation of the NF‐κB inflammatory pathway is preferentially suppressed later, as evidenced by decreased phosphorylation of IKKβ, IκBα and NF‐κB. IMPDH2 also plays a role in the mitogen‐activated protein kinases (MAPKs) signalling pathway. Inhibition of IMPDH2 significantly suppresses the phosphorylation of p38 MAPK, suggesting that p38 MAPK inflammatory signalling constitutes a critical component of IMPDH2's downstream effects [17]. Although IMPDH2 directly affects GTP production, IMPDH2 activity may also indirectly affect ATP levels due to the close relationship and interconversion between GTP and ATP. When IMPDH2 activity is reduced resulting in decreased GTP synthesis, cells may compensate for the lack of GTP by increasing the conversion of ATP to GTP, thus indirectly affecting ATP levels and distribution [20]. Whereas changes in ATP levels affect the AMPK signalling pathway, immediately following an effect on mTOR. This affects the level of autophagy, which is involved in the inflammatory response. In addition, IMPDH2 can participate in the PI3K/AKT/mTOR pathway [4], and this process can also have an effect on autophagy. It is thus clear that IMPDH2 is involved in the inflammatory response through a series of pathways that can have a pronounced effect on autophagy, and the central station in this process is mTOR. IMPDH2 plays a key role in regulating the inflammatory response by influencing the activity of these pathways. So we infer that this process is related to two factors, energy metabolism affects the production and release of cytokines participates in the signalling pathway. On the other hand, IMPDH2 may directly participate in the signalling pathway as an upstream signalling factor.

### Regulate Immune Function

IMPDH2 exhibits activation in immune cells and is associated with drug resistance, making it a potential target for immunosuppressive therapies [21]. Mycophenolic acid (MPA), the active metabolite of MMF, reversibly inhibits IMPDH, an enzyme in the purine synthesis pathway essential for guanosine production and proliferation of lymphocytes [22]. Considering the key role of IMPDH2 in cellular metabolism and proliferation, and its high expression in immune cells, it can be hypothesised that IMPDH2 may influence immune response and immunoregulatory processes by affecting the energy metabolism and proliferation of immune cells. Studies on the precise role of IMPDH2 in immune response and immunomodulation still need to be further developed.

### Role in Ribosomal Stress

The ‘ribosomal stress (RS)‐p53 pathway’ is activated by stressors or genetic alterations that interferes with ribosomal biogenesis and is mediated by several ribosomal proteins (RPs), such as RPL11 and RPL5, which inhibit MDM2 and activate p53 [23]. Previous studies showed that inhibition of IMPDH2 leads to p53 activation through RS. This is because inhibition of IMPDH2 decreases cellular GTP levels [24]. By inhibiting cellular IMPDH2, the level of GTP is reduced, which in turn reduces the level of nucleostemin (NS), which leads to the activation of RS and p53, suppressing tumour growth [15]. Thus, IMPDH2 plays an important role in ribosomal stress.

## Associations of IMPDH2 Disorders and Pathologies

The studies presented above underscore the importance of IMPDH2 in various regulatory pathways at both cellular and molecular levels, supporting the hypothesis that dysregulation of IMPDH2 may be a hallmark of multiple diseases characterised by disruptions in these pathways. Altered expression levels of IMPDH2 impact multiple organ systems and are associated with a variety of diseases. Here, we briefly explore the relationship between the physiological functions of IMPDH2 and its proposed or confirmed dysfunction in diseases (Table 1).

**TABLE 1:** Clinical and therapeutic relevance of IMPDH2 expression and function.

Disease or model system	Observation of clinical or therapeutic significance
Neurodegenerative and neuropsychological diseases	
Early neurodevelopmental disorders	Associated mutations in and around the structural domain of Bateman in IMPDH 2 allow for defects in GTP regulation that disrupt the balance of the purine pool in the nervous system, leading to neurodevelopmental disorders [25].
Neurodevelopmental disorder mutation	Disruption of IMPDH 2‐L245 P filament assembly reduces IC 50 of GTP with therapeutic potential [26].
Myodystony	In cells following mitosis in the central nervous system, low expression of IMPDH 2 allows reduced guanine and dopamine synthesis, leading to dystonia and tremor in children or adolescents [27].
Normal development of neural crest derivatives	Deficiency of Impdh2 in early neural crest results in developmental defects in a variety of neural crest derivatives [28].
Neuron damage	Inhibition of IMPDH2 reduces activation of microglia and astrocytes as well as cytokine production after acute neuronal injury, thereby reducing GTP levels and neuroinflammatory effects promoting neuronal repair and regeneration [29].
Neuroinflammation	Cys140 of the Betamann structural domain in IMPDH2 is selectively bound by the small molecule sappanone A for anti‐neuroinflammatory therapy [17].
Neuroglioma	More IMPDH2 shows better chemosensitivity and may serve as a molecular indicator of prognosis for glioma treatment [30].
Mental disease	IMPDH2 may serve as an actionable novel drug target for the treatment of psychiatric systemic disorders [31].
Musculoskeletal system disorders	
Osteoporosis	Impdh2 deficiency inhibits osteoclastogenesis via mitochondrial oxidative phosphorylation and attenuates ovariectomy‐induced osteoporosis [32]. The IMPDH2 inhibitor, MMF, causes an increase in rankl, which induces osteoclastogenesis and leads to osteoporosis in SLE patients [33]. IMPDH2 are mediated by c‐Myc to affect osteoclast metabolism, leading to osteoporosis [34].
Immune system disorders	
Immune response	Expression of IMPDH2 in pre‐transplant CD4 cells may be an indicator of immune activation [35]. Induction of IMPDH2 and nucleolus expansion are critical for ebvirus transformation of B cells [36]. Measurement of pre‐transplant IMPDH2 levels may help predict individual drug responsiveness and thus improve clinical management of MMF‐treated patients [37].
Cancer	
Osteosarcoma cell	Highly expressed IMPDH2 has been shown to be associated with radiation resistance and chemoresistance, it upregulates anti‐apoptotic proteins, leading to inhibition of the mitochondrial apoptotic signalling pathway [38]; and directly increase its resistance to chemotherapy [39]; significantly shortening the overall survival and disease‐free survival [40]. IMPDH2 can be used for stratification of low‐risk and high‐risk groups in patients with osteosarcoma [41].
Colorectal cancer cell	IMPDH2 may be a protein biomarker and new therapeutic target for colorectal cancer [42] acceleration of cell cycle transitions through activation of PI3K/AKT/mTOR and PI3K/AKT/FOXO 1 pathways [4]. Vitexicarpin exhibits anticancer activity by directly binding to IMPDH2 and promoting c‐Myc ubiquitination by disrupting the interaction between IMPDH2 and c‐Myc, leading to epithelial‐mesenchymal transition (EMT) inhibition [43]. Produce a high prediction in differentiating between sensitive and non‐sensitive patients treated with cetuximab [44]. Activate of impdh2‐mediated purine metabolism by affecting the Wnt/β‐catenin signalling pathway promotes oxaliplatin resistance [45]. A novel, selective, competitive IMPDH2 inhibitor, berberrubine, significantly reduces human colorectal cancer cell growth in a dose‐dependent manner [46].
Oesophageal carcinoma cell	IMPDH2 signalling is inhibited by Fv‐LDP‐D3, resulting in inhibition of oesophageal cancer cell proliferation while improving therapeutic efficacy [47].
Gastric cancer cell	Increased sensitivity of multidrug‐resistant gastric cancer cells SGC7901/VCR to methotrexate by decreased ZNRD1 activity through inhibition of IMPDH2 [48].
Hepatocellular carcinoma cell	Overexpression of IMPDH2 can be used to predict OS and PFS in HCC patients; expression levels are a potential biomarker for identifying poor differentiation and a useful predictor of poor prognosis in hepatocellular carcinoma patients after curative hepatectomy [49]; enhance the phosphorylation and transcriptional activity of JunB [50].
Pancreatic cancer cell	IMPDH2 expression is dependent on AFF4 binding to PAX2 and promotes proliferation of PDAC cells [51].
Lung carcinoma cell	ASCL 1 Low SCLC with high levels of MYC are more sensitive to IMPDH2 inhibition in vivo [52]. IMPDH 2 promotes NSCLC cell proliferation, invasion, migration and EMT through activation of the Wnt/β‐catenin signalling pathway [53]. Genistein promotes apoptosis of lung cancer cells through the IMPDH2/AKT1 pathway [54]. Direct binding of FANCI to IMPDH 2 reduces IMPDH 2 degradation and promotes activation of la MEK/ERK/MMPs signalling pathway [55].
Renal carcinoma cell	Overexpression of IMPDH2 can serve as a biomarker for the diagnosis of kidney cancer as well as a potential therapeutic target [56].
Prostate cancer cell	IMPDH2 interacts with circPFKP to promote guanine nucleotide production and PCa cell proliferation [57]. IMPDH2 may be considered a potential target for PCa therapy using siRNA approaches alone or in combination with other pharmacological strategies [58]. IMPDH2 is a promising candidate biomarker for patients with advanced PCa and those at high risk of progressing to advanced PCa [59]. Targeted Inhibition of IMPDH2 synthesis affects nucleolar stress, therapeutic response and metabolism in Prostate Cancer [60]. Enhanced IMPDH2 expression may promote tumour metastasis and advanced tumour progression in prostate cancer patients [61].
Bladder cancer cell	LncRNA UCA 1 promotes the binding of TWIST 1 to the IMPDH 2 promoter region, which promotes the transcription of IMPDH 2 and increases the proliferation, migration and invasion of bladder cancer cells [62].
Breast cancer cell	Novel, non‐covalent selective inhibitor, shikonin, induces growth arrest and apoptosis in TNBC cell lines through direct inhibition of IMPDH 2 and may be a potential therapeutic candidate for triple‐negative breast cancer [63]. IMPDH2 is highly expressed in breast cancer and predicts poor prognosis [64].
Nasopharyngeal carcinoma cell	High expression of IMPDH2 suggests a poorer prognosis for patients with nasopharyngeal carcinoma [65].
Ovarian carcinoma cells	IMPDH2 expression in ovarian cancer as a potential prognostic biomarker [66].
Cervical cancer cells	Drug inhibitor oxymatrine combined with chemotherapy targeting IMPDH2 may be a promising means to overcome chemotherapy resistance [67].
Leukaemia cell	Inhibition of IMPDH2 induces activation of TLR signalling and up‐regulation of VCAM 1 with therapeutic potential for MLL‐AF 9‐driven AML [68]; and inhibites the proliferation of leukaemia K562 cells [69].
Melanoma cell	Screening of assembled filamentous structures of IMPDH2 helps to distinguish melanoma from nevi and to understand the malignancy of tumours [70].
Lymphoma cell	IMPDH2 induces lymphoma progression through activation of the PI3K/AKT/mTOR signalling pathway [71, 72].
Malignant glioma cells	Up‐regulation of IMPDH2 affects abnormal nucleolus function and increased anabolic processes in GBM and is a major cause of glioma formation [73]. Increasing the therapeutic efficacy of temozolomide in malignant glioma by inhibiting IMPDH2 [74].
Urinary system disorders	
Renal fibrocyte fibrosis	Inhibition of IMPDH2 attenuates renal fibroblast fibrosis [75].
Renal ischemia–reperfusion injury	MGC reduces renal I/R injury and promotes restoration of reperfusion in rats by inhibiting renal IMPDH2 activation and decreasing recruitment of lymphocytes and monocytes to sites of inflammation [76].
Other diseases and conditions	
Lipometabolism	Impdh2 inhibitor MMF effectively inhibits high‐fat feeding‐induced white adipose tissue expansion [77]. Impdh2 deficiency in adipocyte precursors limits white adipose tissue expansion and enhances metabolic health in over nutrition [78].

### Neurodegenerative and Neuropsychological Diseases

Recent studies have shown that multipoint mutations in the human IMPDH2 isoform are closely related to dystonia and other neurodevelopmental disorders. Specific mutations in the IMPDH2 protein, particularly around the Bateman structural domain, impair its regulation by GTP, leading to an imbalance in the purine pool within the nervous system and contributing to neurodevelopmental diseases [25]. One significant mutation, IMPDH2‐L245P, lowers the IC50 of GTP and suggests therapeutic potential for neurodevelopmental disorders [26]. Deletion of IMPDH2 in the early neural crest leads to defects in a variety of neural crest derivatives. IMPDH2‐mediated guanine nucleotide synthesis is crucial for the normal development of the ENS and other neural crest derivatives [28]. Studies suggest that IMPDH2 gene degradation may result in protein deficiencies in patients, implicating it in dystonia. Low expression of IMPDH2, resulting in a low nucleotide pool, allows for reduced guanine and dopamine synthesis, leading to dystonia and tremor in children or adolescents [27].

Acute central nervous system (CNS) injuries often result in perpetual functional impairments. In addition to the primary injury, acute inflammation and subsequent low‐grade chronic neuroinflammation in later stages are important in influencing the progression of the injury, its complications, and prognosis [79, 80]. After screening, the small molecule saponin A (SA) targeted to IMPDH2, offering potential for anti‐neuroinflammatory therapies [17]. Mycophenolate mofetil (MMF), a selective inhibitor of IMPDH, applied within a specific time window after neuronal injury, reduces activation and cytokine production in microglia and astrocytes after acute neuronal injury through inhibition of IMPDH2 [29]. This offers great potential for the development of well‐tolerated therapeutic agents against neuroinflammatory conditions based on the IMPDH2 protein.

In addition, IMPDH2 is strongly associated with neurological tumour diseases. Gliomas are the most common primary tumours of the CNS [81]. Patients with a higher IMPDH1/IMPDH2 ratio demonstrated poorer outcomes. In the presence of IMPDH2 dominance, the imbalance in the cellular GTP/GDP ratio impairs DNA damage repair capacity and makes cells more sensitive to Temozolomide (TMZ) [30].

Medication options for treating mental disorders are limited, and most medications currently in use are ineffective in a significant number of patients. Using transcriptomic data, they identified IMPDH2 as a potential therapeutic target for depression, offering a promising opportunity for drug repurposing in the treatment of psychiatric disorders [31].

### Musculoskeletal System

Previous studies have demonstrated that IMPDH2 positively regulates bone resorption. Deficiency of IMPDH2 in mouse myeloid cells inhibited osteoclast generation and increased bone mass. In addition, IMPDH2 deficiency impaired osteoclast mitochondrial function and attenuated ovariectomy‐induced bone loss [32]. In addition, IMPDH2 and CTPS are mediated by c‐Myc to affect osteoclast metabolism, leading to osteoporosis [34]. However, it has also been demonstrated that osteoporosis can occur in patients with systemic lupus erythematosus (SLE) secondary to medication‐induced effects and chronic inflammation, and that SLE patients have significantly impaired osteoclast differentiation. Treatment with the IMPDH1/2 inhibitor MMF promoted osteoclastogenesis and reversed this impaired differentiation [33].

### Immune System Disorders

Immunosuppression is essential for the survival of both the recipient and the graft after transplantation. Advances in surgical and immunosuppressive therapies have greatly improved the prognosis of organ transplant recipients in the past few decades [82]. Mycophenolic acid (MPA), the active metabolite of MMF, reversibly inhibits IMPDH; guanosine production and proliferation of lymphocytes are inhibited [22]. Over the past few decades, IMPDH2 has emerged as a promising drug target for the treatment of autoimmune diseases, and IMPDH inhibitors appear to be an effective immunosuppressant in clinical trials [83].

In vitro growth transformation of primary B cells by ebvirus (EBV) is the first step in the development of post‐transplant lymphoproliferative disorders (PTLD). The inhibitor MPA acts on IMPDH2 to block the growth transformation of primary B cells by EBV, resulting in smaller nucleoli, nuclei and cells that inhibit PTLD [36]. Given the action of MPA on IMPDH and the potential to monitor MPA pharmacodynamics by measuring IMPDH activity, understanding changes in IMPDH2 expression during immunosuppression is crucial. IMPDH2 expression was significantly down‐regulated in whole blood and reticulocytes but increased in CD4 cells before transplantation and after immunosuppressive therapy, and pre‐transplantation expression of IMPDH2 in CD4 cells may be an indicator of immune activation [35]. Measuring pre‐transplant levels of IMPDH1/2 could also help predict patient responsiveness to MMF, optimising its clinical management [37].

### Cancer

The imbalance in the metabolism of purine nucleotides is a major factor in cancer development and progression. Tumour cells exhibit strong activation of the guanine nucleotide synthesis pathway, leading to an increase in metabolite levels. This provides more energy for physiological processes, such as signal transduction, energy transport and protein synthesis, thereby driving the malignant behaviour of cancer cells. Aberrant expression of IMPDH 2 causes abnormal guanine nucleotide synthesis and affects its progression. Similar effects have been seen in a variety of tumours, such as oesophageal cancer, hepatocellular carcinoma, pancreatic ductal adenocarcinoma and prostate cancer [30, 40, 47, 49, 56, 60].

**FIGURE 3:**
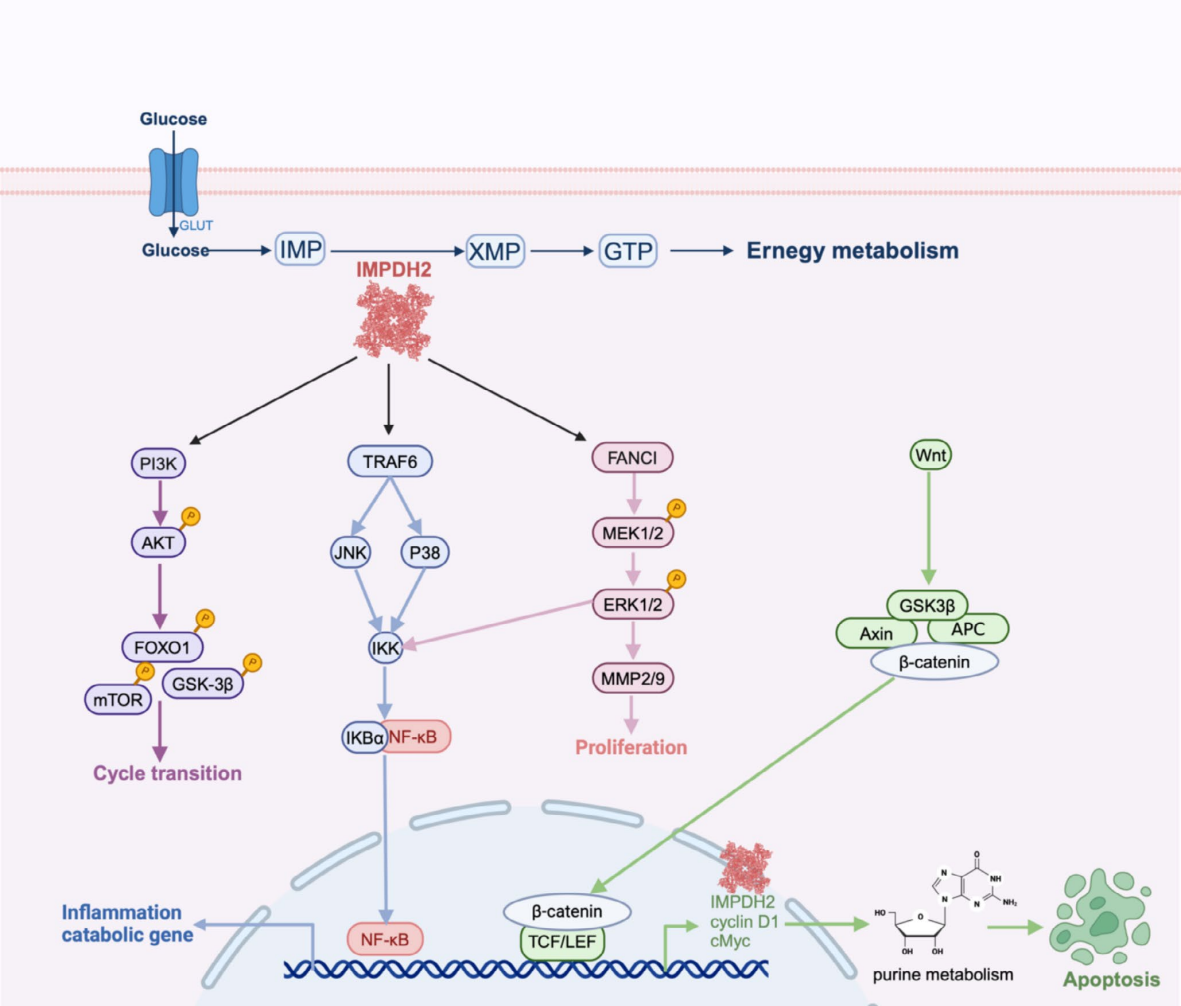
Energy metabolism and signalling pathways involved in IMPDH2. (A) IMPDH2 is an essential enzyme involved in the de novo synthesis of guanine nucleotides. (B) Accelerate cell cycle transition through activation of the PI3K/AKT/mTOR and PI3K/AKT/FOXO 1 pathways. (C) Involved in NF‐κB signalling pathway, affects inflammatory response. (D) Binds FANCI and participates in the MEK/ERK/MMP pathway thereby affecting proliferation. (E) The Wnt/β‐catenin/IMPDH2 mediated purine metabolism regulating apoptosis.

Since then, research has shown that IMPDH2 is also related to a variety of signalling pathways. For example, it can influence colorectal cancer by participating in the PI3K/AKT/mTOR and PI3K/AKT/FOXO 1 signalling pathways [4]. It promotes proliferation and migration in non‐small cell lung cancer by activating the Wnt/β‐catenin signalling pathway [53] and enhances IMPDH2‐mediated purine metabolism in colorectal cancer cells, thereby inhibiting caspase‐dependent apoptosis [45]. It impacts lung adenocarcinoma by participating in the MEK/ERK/MMPs signalling pathway [55], affects leukaemia through TLR signalling [68] and interferes with osteosarcoma by inhibiting the mitochondrial apoptotic signalling pathway [38] (Figure 3).

Furthermore, IMPDH2's receptor‐ligand interactions and its involvement in protein complexes have been linked to cancer cell proliferation. In malignant gliomas, IMPDH2 expression is downregulated by inhibiting the interaction between IMPDH2 and ADP‐ribosylated factor‐like protein 13B(ARL13B), leading to the inhibition of purine nucleotide synthesis pathways. This, in turn, enhances the therapeutic efficacy of temozolomide [74]. Knockdown of circPFKP in prostate cancer cells inhibits cell proliferation [57]. In lung adenocarcinoma cells, inhibiting IMPDH2 overexpression, reducing FANCI‐IMPDH2 binding, promoting IMPDH2 degradation and blocking the MEK/ERK/MMPs pathway [55]. Vitexicarpin breaks the bond between IMPDH2 and c‐Myc, thereby promoting c‐Myc ubiquitination and inhibiting epithelial‐mesenchymal transition (EMT) [43]. Overexpression of IMPDH2 activates JunB phosphorylation, leading to enhanced JunB transcriptional activity [50]. Across cancer cell lines, IMPDH2 overexpression has a similar effect on cell survival through interactions with proteins.

IMPDH2 can be used as a prognostic indicator of disease. IMPDH2 expression stratifies osteosarcoma patients into high‐risk and low‐risk groups, with higher expression significantly reducing both overall and disease‐free survival [40, 41]. IMPDH2 can also be used as a promising candidate biomarker for patients with advanced and progressed to advanced high‐risk prostate cancer [59]. In ovarian cancer cells, high expression of IMPDH2 can be found to serve as a prognostic marker [66]. IMPDH2 is overexpressed in breast cancer and predicts poor prognosis [64].

IMPDH2 has been selected as a marker of drug resistance in several cancers; potential aberrant activation or accumulation of IMPDH2 may be the basis for the establishment of chemoresistant cell populations. Activation of IMPDH2‐mediated purine metabolism by the Wnt/β‐catenin signalling pathway promotes oxaliplatin resistance by inhibiting caspase‐dependent apoptosis in colorectal cancer [45]. In osteosarcoma cells, overexpression of IMPDH2 has been shown to be associated with radiation and chemotherapy resistance, and high chemoresistance upregulates anti‐apoptotic proteins [38, 39]. The interaction between IMPDH2 and ARL13B enhances purine remedy, thereby increasing resistance to temozolomide [74]. In multidrug‐resistant gastric cancer cells (SGC7901/VCR), elevated IMPDH2 expression leads to increased ZNRD1 activity and decreased sensitivity to methotrexate [48]. High expression of IMPDH2 signalling promotes oesophageal cancer cell proliferation while elevating cellular resistance [47].

IMPDH2 may serve as a potential molecular target for therapy. In triple‐negative breast cancer cells, treatment with comfreyin shikonin has been reported to lead to downregulation of IMPDH2 expression. Inhibition of IMPDH2 increases growth arrest and apoptosis of TNBC cells, suggesting that IMPDH2 may be a therapeutic target [63]. The oxytetracycline in combination with chemotherapy targeting IMPDH2 may be a promising means of overcoming chemoresistance in cervical cancer [67]. Berberrubine, a novel, selective, competitive inhibitor of IMPDH2, has shown a 15‐fold greater selectivity for IMPDH2 over IMPDH1, significantly reducing colorectal cancer cell growth in a dose‐dependent manner [46]. Furthermore, vitexicarpin exhibits anticancer activity by directly binding to IMPDH2, leading to epithelial‐mesenchymal transition (EMT) inhibition [43]. In multidrug‐resistant gastric cancer, downregulation of ZNRD1 activity via IMPDH2 inhibition enhances sensitivity to methotrexate [48]. In oesophageal cancer, inhibiting IMPDH2 signalling suppresses cell proliferation and improves treatment efficacy [47]. In gliomas, inhibiting the interaction between IMPDH2 and ARL13B enhances the therapeutic efficacy of temozolomide [74]. Collectively, the studies suggest that IMPDH2 could be a potential molecular target for therapeutic intervention.

### Urinary System Disorders

The direct role of IMPDH2 in the pathology of urinary tract diseases remains largely unknown. Transforming growth factor‐β1 (TGF‐β1) plays a major role in fibrosis, and TGF‐β1‐induced fibrogenesis is an intracellular metabolic dysfunction associated with chemokine‐mediated inflammation [84]. Additional insights into the biological background of TGF‐β1‐induced fibrosis are characterised by dysregulation of metabolic pathways [85]. IMPDH2 inhibitors provide therapeutic benefits for fibrosis inhibition [75, 86].

Renal ischemia/reperfusion (I/R) injury poses a substantial risk of mortality and morbidity during renal surgery, but current medications are generally characterised by high doses or prolonged use due to the lack of drugs that specifically target the kidney [87]. In a previous study, it was described that a novel mycophenolic acid‐glucosamine coupling (MGC) which reduces renal I/R injury and promotes restoration of reperfusion recovery by inhibiting renal IMPDH2 activation and depleting guanosine ribonucleotides [76]. This IMPDH2‐targeted approach may provide a more effective substitution to existing combination treatment.

### Other Diseases and Conditions

The balance between hypertrophic growth of mature adipocytes and adipogenesis is critical for managing the metabolic stability of white adipocytes under nutrient overload. Previous studies revealed increased expression of IMPDH2 during the adipogenic both in vivo and in vitro, with Xmp enhancing lipogenic potential by promoting mitotic clonal expansion (MCE). It has been shown that the IMPDH inhibitor MMF effectively blocks high‐fat diet (HFD)‐induced WAT expansion [77]. Meanwhile, the knockdown of IMPDH2 in APCs significantly reduced HFD‐induced neoadipocyte counts but did not affect adipocyte size [78]. Importantly, targeting IMPDH2 offers a promising therapeutic approach for combating obesity.

## Conclusion and Perspectives

An increasing body of evidence indicates that IMPDH2 is a multifunctional protein involved in a wide range of physiological processes and disease states. The unique structure, multiple subcellular localizations, and tissue‐specific expression patterns contribute significantly to the complexity of its functions. Recent studies also highlight its critical role in a range of pathological conditions. IMPDH2 affects energy metabolism, inflammatory response, immune response, autophagy and ribosomal stress, plays a pivotal role in cell proliferation, differentiation, invasion, migration, tumorigenicity and epithelial mesenchymal transition. Indeed, existing literature has linked aberrant IMPDH2 expression to abnormal bone and cartilage development, cancer, neurodegeneration and immune system disorders. It is closely related to grading, treatment and prognosis. Targeted regulation of IMPDH2 could improve the progression of multiple diseases. However, current small molecule drugs lack selectivity, resulting in many adverse reactions that limit their clinical use. Targeted drugs based on IMPDH2 will become the focus of our future research.

## Author Contributions

Z.L. wrote the original draft and review and editing. A.Y., Y.Z. and J.N. contributed to visualisation. Y.Z. and W.L. contributed to project administration. R.L., S.W. and J.G. contributed to supervision, project administration and funding acquisition. All authors contributed to critical revision of the manuscript for important intellectual content.

## Conflicts of Interest

The authors declare no conflicts of interest.

## Data Availability

Data sharing is not applicable to this article as no new data were created or analyzed in this study.
